# The Modulating Effect of Personality Traits on Neural Error Monitoring: Evidence from Event-Related fMRI

**DOI:** 10.1371/journal.pone.0042930

**Published:** 2012-08-22

**Authors:** Zrinka Sosic-Vasic, Martin Ulrich, Martin Ruchsow, Nenad Vasic, Georg Grön

**Affiliations:** 1 Department of Psychiatry and Psychotherapy III, University Hospital of Ulm Leimgrubenweg, Ulm, Germany; 2 TransferCenter for Neuroscience and Learning, University Hospital of Ulm, Beim Alten Fritz, Ulm, Germany; 3 Department of Psychiatry, Christophsbad Hospital Göppingen Faurndauer, Göppingen, Germany; 4 Section for Neuropsychology and Functional Imaging, University of Ulm Leimgrubenweg, Ulm, Germany; Bellvitge Biomedical Research Institute-IDIBELL, Spain

## Abstract

The present study investigated the association between traits of the Five Factor Model of Personality (Neuroticism, Extraversion, Openness for Experiences, Agreeableness, and Conscientiousness) and neural correlates of error monitoring obtained from a combined Eriksen-Flanker-Go/NoGo task during event-related functional magnetic resonance imaging in 27 healthy subjects. Individual expressions of personality traits were measured using the NEO-PI-R questionnaire. Conscientiousness correlated positively with error signaling in the left inferior frontal gyrus and adjacent anterior insula (IFG/aI). A second strong positive correlation was observed in the anterior cingulate gyrus (ACC). Neuroticism was negatively correlated with error signaling in the inferior frontal cortex possibly reflecting the negative inter-correlation between both scales observed on the behavioral level. Under present statistical thresholds no significant results were obtained for remaining scales. Aligning the personality trait of Conscientiousness with task accomplishment striving behavior the correlation in the left IFG/aI possibly reflects an inter-individually different involvement whenever task-set related memory representations are violated by the occurrence of errors. The strong correlations in the ACC may indicate that more conscientious subjects were stronger affected by these violations of a given task-set expressed by individually different, negatively valenced signals conveyed by the ACC upon occurrence of an error. Present results illustrate that for predicting individual responses to errors underlying personality traits should be taken into account and also lend external validity to the personality trait approach suggesting that personality constructs do reflect more than mere descriptive taxonomies.

## Introduction

Personality traits are defined as habitual patterns of thoughts, emotions and behavioral tendencies which are considered to be relatively stable over time, to differ among individuals, and to influence behavior. One of the most influential trait theories is the Five Factor Model (FFM) of personality [1] describing the five most common and overarching personality traits, the so-called “Big Five”, namely Neuroticism, Extraversion, Openness to Experience, Agreeableness and Conscientiousness. Recent imaging studies on the neurobiological underpinnings of Big Five traits have mainly focused on Neuroticism and Extraversion [2]. Using voxel-based morphometry, Neuroticism was reported to be negatively associated with gray matter (GM) concentration in the right amygdala, while Extraversion was positively associated with GM concentration in the left amygdala [3]. Another study reported a relationship between Extraversion and anatomical size of the orbitofrontal cortex (OFC) [4]. Associations between sizes of specific brain areas and all traits of the FFM have recently been investigated in a sample of 116 healthy adults [5]. In this study, Agreeableness covaried with GM volume in the posterior superior temporal sulcus and posterior cingulate cortex, Extraversion and Neuroticism covaried with volume of brain regions involved in the processing of reward, punishment, and negative affect, and Conscientiousness covaried with increased volume of the left middle frontal gyrus known to be involved in planning and volitional control of behavior. Investigating the functional connectivity of brain regions during the resting state of the brain (no functional challenge), Extraversion was shown to modulate connectivity between brain regions involved in the processing of reward and motivation, while Neuroticism was associated with resting state connectivity between brain regions involved in self-evaluation and fear [6]. In the same study, Openness to Experience was correlated with increased connectivity in the dorsolateral prefrontal cortex (DLPFC).

Functional magnetic resonance imaging (fMRI) studies have shown that Extraversion and Neuroticism modulate neural activity in prefrontal and subcortical brain regions during affective processing. In a mood induction paradigm, increased activation of amygdala in response to happy faces was positively correlated with Extraversion [7], while Neuroticism increased amygdala activation upon negative facial expressions [8]. Similarly, decreased functional connectivity between the anterior cingulate cortex (ACC) and the amygdala during processing of negative emotional facial expressions was correlated with Neuroticism [9]. Based on these previous findings on significant brain-trait relationships, in the present study we sought to investigate the modulating effect of Big Five personality traits on neural activity asssociated with error signaling. This specific process, which indicates an actual incongruence between an intended and performed action [10], appears as a necessary prerequisite to initiate ensuing error related processing, as for example post-error adjustments of neural resources to re-improve performance rates [11]. The neural system involved in error signaling has been intensively investigated in electrophysiological [12] and fMRI studies [13]. These studies identified the ACC together with the adjacent pre-supplementary motor area (pre-SMA) [14], [15], [16], [17] as core neural substrates of error processing. Additionally, previous studies have reported increased error related activation of the lateral inferior frontal cortex encompassing the frontal operculum and parts of the anterior insular [17], [18], [19], [20], [21], [22], [23].

Electrophysiological studies have already examined the modulation of error signaling by individual expressions of different personality characteristics such as sociality [24], impulsiveness [25], negative emotionality [26] or general anxiety [27]. For modulating influences of the Big Five personality traits, a recent event-related potential study indicated that individuals scoring high on Neuroticism showed increased error-related negativity (ERN) in response to errors during an arrow version of the Flanker Task [28]. Assessing the personality trait “persistence” as an analogue to the Big Five trait Conscientiousness, a less pronounced decrease of the ERN after reward manipulation was observed in more conscientious subjects [29]. This finding is consistent with those from a previous study showing that individuals with greater individual expressions of Conscientiousness demonstrated smaller motivation-related variation in error signaling [30]. In contrast, to date, no functional MRI studies have been conducted investigating the association between Big Five personality traits and error processing. However, Horn and colleagues investigated in a fMRI study the impact of impulsivity on error processing during a GoNogo-task. Many trait theorists consider Extraversion and Impulsivity to subsume similar emotional, cognitive, and behavioral patterns sharing the same biological underpinnings [31], [32]. The authors identified an association between Eysenck's impulsivity score on a single factor and the engagement of right orbitofrontal cortex in impulsive individuals [33].

Our intention in the present investigation was to explore, if, and to which extent, Big Five personality traits modulate cerebral activation during a basic, well-defined, circumscribed, and already well-investigated executive process such as error processing. Error signaling is a fundamental human cognitive function, which provides important evaluative information by indicating incongruence between intentions and actions, and therefore is crucial for adjustment of behavior [10]. However, not all individuals are equally effective in behavioral, cognitive, and affective processing of errors, with some committing more errors than others, some avoiding errors more than others, and some others being more bothered by the occurrence of an error than other subjects. Thus, while an interplay between error processing and personality traits seems apparent on a behavioral way, it seems inviting to investigate such an interplay at a neurofunctional level.

To further explore neurobiological implications of personality traits in the domain of error processing, we employed a well-established Eriksen-Flanker/GoNogo-task during event-related fMRI. In two previous studies [34], [35] this task has been shown to produce robust individual error signals. Individual heights of this error signal were tested on their correlation with individual expressions of all of the Big Five personality traits. Since studies so far either reported significant findings for a subset of these traits, or were using different tasks during fMRI, or employed electrophysiological signals, in the present study specific expectations on anatomical location, extent and directions of correlations for each of the five different traits could not be formulated. In order to avoid biasing the neural process of interest, testing on significant correlations was locally constrained to the neural network reliably involved in error signaling at the group level. Hence, for some of the brain regions discussed above no correlations were to expect since these regions have not been shown to play a major role in the neural “error matrix”. Conversely, some of the psychological mechanisms involved in each of the FFM's traits will most likely not be triggered by error signaling so that not all traits were expected to correlate. Specifically, Agreeableness has been related to psychological mechanisms involved in social information processing which however was not tested by the present task (but kept included to test on discriminant validity). On the other side, Openness to Experience involves psychological mechanisms of flexible and effective information processing which in turn can be linked to prefrontal functions of attentional control [30] that align with the present task. Even stronger correlations with error signaling were expected for Extraversion and Neuroticism given previous empirical results, and since both traits have strong relations with an individual's sensitivity to reward and punishment [5] which in turn are evident characteristics of committing errors. Similarly, a stronger modulation effect was expected for Conscientiousness since this trait reflects goal-directed behavior and a strong focus on task accomplishment [1], mediated by resistance against inadequate response tendencies, which was tested by the present task and relates to prefrontal functioning.

## Materials and Methods

### Subjects

27 healthy subjects (13 females and 14 males) recruited via advertisements were studied. Upon invitation all subjects were interviewed by an experienced psychologist (ZS-V) and none of the subjects had any signs or history of neurological or psychiatric diseases. All subjects were right-handed as assessed by the Edinburgh Handedness Inventory [36]. The entire group of subjects had a mean of 24.30 (SD: 4.13; range: 20–35) years of age and a mean of 12.81 (SD: 0.98; range: 8–13) years of school education. Males and females did not differ significantly on years of age [t(25) = 0.85; p = 0.404)] or on years of school education [t(25) = −1.04; p = 0.309)]. All subjects were paid for their participation. The project was approved by the Institutional Review Board of the University of Ulm, Germany. Written informed consent according to the Declaration of Helsinki was obtained from each individual subject.

### Psychometric Measurement of Personality

Subjects were investigated with the German version of the NEO-Personality Inventory - Revised [37]. The NEO-PI-R consists of 240 items answered on a five point scale, ranging from “strongly disagree” to “strongly agree”. The following expressions represent representative items for each of the five dimensions (re-translated from the German version of the questionnaire). *Neuroticism:* “I easily get scared”; *Extraversion:* “I love to be surrounded by other people”; *Openness to Experience:* “I love to resolve problems or tricky tasks”; *Agreeableness:* “I prefer cooperation to others to competition with them”; *Conscientiousness:* “I am working hard to achieve my goals”. Internal consistencies of this questionnaire range from 0.75 (Agreeableness) to 0.83 (Conscientiousness), and test-retest reliability over 6 years was reported to range from 0.63 (Agreeableness) to 0.83 (Neuroticism and Openness to Experience).

### fMRI-Paradigm

**Figure 1:**
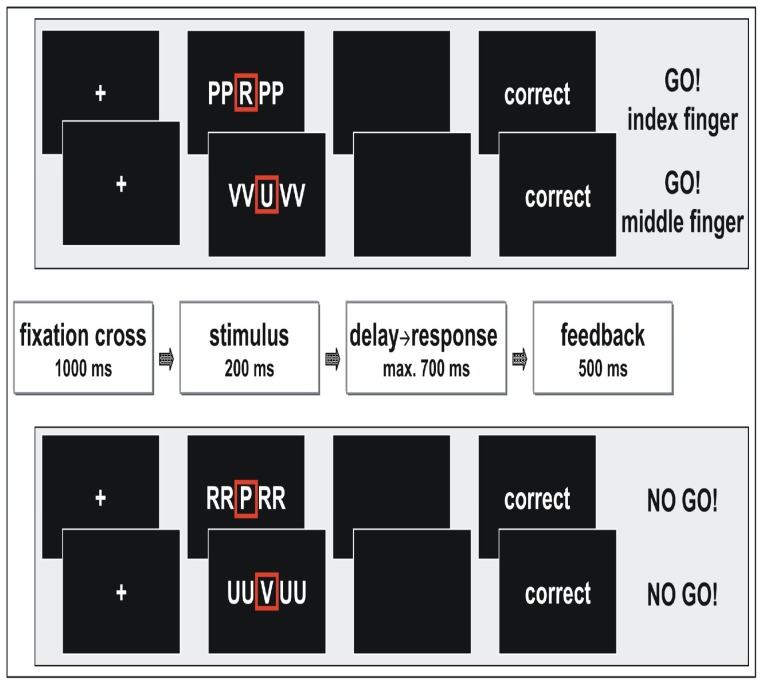
Combined Eriksen Flanker and GoNoGo fMRI paradigm, exemplary shown for all four incongruent trials. After the fixation period, one of eight possible letter strings, either congruent or incongruent, appeared on a black screen. Subjects were instructed to give a right hand index finger response, if the target letter was a “R”, to give a right hand middle finger response, if the target letter was an “U”, or to withhold response in case of appearance of target letters “P” or “V”. One of three possible feedbacks (“correct”, “wrong”, “faster”) about the subjects' response was given after a defined delay following response (in this example “correct”). *Upper panel*: Go trials. *Lower panel*: NoGo trials.

We employed a combined Go/NoGo-Eriksen-Flanker-paradigm [38] established in several electrophysiological studies on error monitoring [25]. Five-letter strings were created from the letters R, U, P, and V with the action relevant target always mid-standing. During Go-trials, subjects were asked to respond with their right index finger on a two-button box to the target letter R, and with their middle finger to the target letter U (for the rationale of this additional two-alternative forced choice condition, see Supplementary Data). In NoGo-trials subjects should withhold a response upon appearance of the letters P or V. Target and flanker stimuli were combined either congruently or incongruently. In congruent trials, all five letters were the same. In incongruent Go-trials, Go-targets were flanked by visually similar NoGo-target letters (e.g. VVUVV). In incongruent NoGo-trials the central NoGo-target was flanked by visually similar Go-targets (e.g. UUVUU; Figure 1). Each trial ended with the presentation of feedback according to the subjects' performance and which was presented for 500 ms (see also below). Presentation of the experimental task was run via ERTS software (“Experimental Run Time System”). Visual stimuli were centrally presented by means of MR compatible video goggles. Reaction times and correctness of subjects' responses on each trial were automatically registered by a standard personal computer that also controlled the sequence of trials.

Each combination of the factors condition (Go, NoGo) and type (congruent, incongruent) contained 66 trials resulting in a total of 264 trials. The task was implemented in a rapid event-related fMRI design. One trial lasted 1.9 seconds and was triggered by an optical signal emitted from the MR scanner with the beginning of each TR. To ascertain continuous sampling of the hemodynamic response function, a jitter was inserted after the trigger signal randomly distributed in multiples of slice acquisition time. Mean inter-trial interval was 3.01 seconds. The average stimulus-onset asynchrony for events of the same combination of condition by type (e.g. incongruent NoGo) was 19.5 seconds. The entire task duration was 22 minutes. Subjects got acquainted to this challenging task during training sessions at the morning of the test day. The training version was different from the test version with respect to trial sequences and number of trials, but identical as regards number and realizations of the conditions. Further, a reaction time (RT) deadline was individually estimated by calculating the average individual reaction time on correct congruent and incongruent Go trials obtained from the training session prior to the fMRI task. From this average a further 15 percent were subtracted. The resulting value in milliseconds served as upper bound of a time window within which Go responses had to be executed during the fMRI task. Whenever subjects' individual reaction times on correct Go trials were above this upper border the ensuing feedback screen contained the German word for “faster”. If Go reactions were correct and within the predefined time limit the feedback was the German word for “correct”. For incorrect Go responses the feedback screen was the German word for “wrong” irrespective of whether reaction times were within or beyond the time limit.

Across subjects individual RT deadlines ranged between 366 ms and 525 ms (mean = 441 ms, SD = 36.7 ms). The rationale behind this was i) to emphasize overall speed in order to obtain sufficient frequencies of erroneous Go and NoGo responses, and ii) to exert control over the speed-accuracy trade off such that correct and false Go responses could be calculated within and above the pre-adjusted time limits.

### Functional Data Acquisition

Imaging data were acquired using a 3.0 Tesla head-only MRI-system (Siemens Magnetom Allegra, Erlangen, Germany). T2*-weighted MR-images were obtained using gradient echo-planar imaging (EPI) in axial orientation along the AC-PC-line. Inplanar matrix size was 64 by 64 pixels (3.6×3.6 mm pixels). The volume consisted of 33 slices (TR = 2200 ms; TE = 39 ms; BW = 3906 Hz/Pixel). Slice thickness was 3 mm with a gap of 0.75 mm. For each session 608 volumes were acquired. The first 10 volumes of each session were discarded to avoid T1 equilibration effects. High resolution T1-weighted anatomical images were obtained using 3D-MPRAGE sequences (BW = 130 Hz/Pixel, TR = 2300 ms, TI = 1.1 s, TE = 3.93 ms, flip-angle = 12°) and at least 160 contiguous slices of 1 mm thickness in sagittal direction (depending on subjects' head sizes).

### Data Analysis

#### Behavioral data analysis

From each subject rates of correct and incorrect responses were computed for the different combinations of the factors *condition* (Go/NoGo), and *type* (congruent/incongruent). In case of Go trials an additional factor *deadline* (within/beyond) was added expressing whether correct and incorrect Go responses had been executed within or beyond the individually predefined time window. Statistical inference of significant differences on error rates was computed using an analysis of variance modeling combinations of the factors above including post-hoc contrasts for comparisons of interest (Newman-Keuls test; p<0.05; see Results). In case of erroneous responses during NoGo trials and omissions during Go trials the effect of factor *type* (congruent/incongruent) was tested by means of paired t-tests for either variable. For all the three analysis above the nominal level of significance was set to level of p<0.05.

Although individual expressions of each of the five dimensions obtained from the NEO-PI-R should demonstrate only weak inter-correlations, if at all, we nevertheless tested this prediction for the present sample in order to identify co-linear regressors. Therefore, inter-correlations among the five NEO-PI-R major domain scores were analyzed using Pearson correlation coefficients (level of significance: p<0.05). To test for relationships between individual expressions of the five NEO-PI-R scores and behavioral performances in the experimental fMRI task again Pearson correlation coefficients were computed (level of significance: p<0.05).

#### Analysis of functional MRI data

Image preprocessing was carried out using Statistical Parametric Mapping (SPM8, Wellcome Department of Cognitive Neurology, London, UK) under MATLAB 7.8 (Math-Works, Natrick, MA). Data from each experimental session were preprocessed including slice timing, realignment and normalization into a standard template (Montreal Neurological Institute, MNI). Normalized images were resliced to 2×2×2 mm^3^ voxels and finally smoothed in space with a three-dimensional 10 mm full-width at half-maximum (FWHM) isotropic Gaussian kernel. For normalization purposes high-resolution individual T1-weighted images were used that had been co-registered to the individual mean EPI obtained during spatial realignment.

Individual event types (see below) were modeled as trains of delta functions at each stimulus onset and convolved with the canonical hemodynamical response function (HRF). The association between each predictor and the experimental voxel time courses was calculated and served as a parameter estimate for the magnitude of neural activity for each event type. A second set of regressors was added to the individual design matrix using the 6 motion parameters acquired during the realignment procedures. During calculation of parameter estimates time series were scaled to a grand mean of 100 over all voxels and volumes within a session. Low frequency drifts were removed via a high pass filter using low-frequency cosine functions with a cut-off of 128 seconds. Model estimation was corrected for serial correlations using a first-order autoregressive model. The different regressors of each individual design matrix were defined by combinations of the factors *condition* (Go/NoGo), *type* (incongruent/congruent), *response accuracy* (incorrect/correct), and *deadline* (within/beyond). Missed Go events (errors of omission) were also modeled. Differentiation between target letters was not considered at this stage of design specification. Subject specific condition effects were computed using signed one-tailed *t*-contrasts, producing a contrast image propagated to the second level analysis.

To account for inter-individual variance and in order to generalize from statistical inferences of condition effects and their relevant contrasts, a random-effects analysis using individual contrast images from the first level analyses was set up. As it will be shown during presentation of behavioral results erroneous responses on congruent Go and NoGo were not consistently produced across subjects (see Results, Table 1). Only for the incongruent NoGo condition all subjects committed errors. Therefore, all analyses reported below were computed for this condition in order to use the entire data set of all 27 subjects for correlation analyses (for results on Go errors, conjoint effects of NoGo and Go errors, and significant positive and negative correlation between the Go error signaling and the NEO-PI-R major domain scores obtained from the reduced model after excluding scales Agreeableness, Extraversion and Openness not showing any significant correlations in the full mode, see Supporting Information S1). For identifying brain activity related to *NoGo errors* a corresponding [incorrect NoGo minus correct NoGo] contrast was formulated as signed t-contrast. To infer a significant main effect at the group level the significance threshold was set to p<0.025 to account for the one-sidedness of this t-contrast in combination with a family-wise error (FWE) correction to account for multiple comparisons.

**Table 1:** Summary statistics of the different error rates (%) during the fMRI task.

Dependent Variable	Error (%)	*p*
*Omissions on Go trials*		
Incongruent	5.2 (8.8); [10]	t(26) = 0.90; *0.374*
Congruent	4.4 (6.4); [9]	
*Incorrect Go trials; in time*		
Incongruent	6.2 (5.7); [3]	*0.940*
Congruent	6.3 (5.9); [4]	
*Incorrect Go trials; delayed*		
Incongruent	4.8 (2.7); [1]	*0.260*
Congruent	3.1 (3.1); [5]	
*Correct Go trials; in time*		
Incongruent	31.8 (12.2)	*0.0002*
Congruent	39.5 (14.8)	
*Correct Go trials; delayed*		
Incongruent	52.0 (15.6)	*0.001*
Congruent	46.7 (17.0)	
*Ommissions on NoGo trials*		
Incongruent	24.9 (14.4)	t(26) = 10.55; *<0.0001*
Congruent	7.5 (9.6); [2]	

Values are means ± SD (standard deviation) in rounded brackets; p values stem from Newman-Keuls post-hoc tests or from paired t-tests; digits in squared brackets denote numbers of subjects who did not produce any erroneous responses on the corresponding condition-by-response type-combination. Only for the incongruent NoGo condition all subjects committed errors.

Furthermore, multiple regression analyses were conducted. Testing on associations between individual expressions of the NEO-PI-R major domain scores and individual neural error signaling on incongruent NoGo trials (as defined above) was performed using a two-step multiple regression analyses due to the observation of substantial inter-correlations between scales in the present sample (see Table 2). Additionally, to discard effects of age, this independent variable was added to the design matrix. In the first step all six regressors (five scales plus age) were included into the full model to test on significant correlations of each scale beyond and above all other scales. Computation of F-contrasts for each scale was explicitly constrained to the statistical map associated with error signaling during incongruent NoGo trials derived from the above group analyses. For this full model an exploratory threshold was set to a level of p<0.001, uncorrected at the voxel level. This more lenient threshold was used to ascertain that possibly relevant correlations were not simply masked out by a too strong statistical threshold. In a second step, only those regressors kept included into a reduced model that had produced significant results within the full model. For this final analysis, however, the statistical threshold was strengthened and set to a family-wise corrected p-value of p<0.05.

**Table 2:** Inter-correlations among NEO-PI-R major domain scores.

Variable	N		E		O		A		C	
**N**	1.00		−0.55	*0.003*	−0.02	n.s.	−0.31	n.s.	−0.51	*0.006*
**E**			1.00		0.46	*0.016*	0.18	n.s.	0.14	n.s.
**O**					1.00		0.29	n.s.	−0.25	n.s.
**A**							1.00		0.11	n.s.
**C**									1.00	
**Age**	−0.11	n.s.	−0.21	n.s.	0.00	n.s.	−0.01	n.s.	0.34	n.s.

N, E, O, A, and C are the following subscales from the NEO-PI-R: N = Neuroticism; E = Extraversion; O = Openness to Experience; A = Agreeableness; C = Conscientiousness. Values are correlation coefficients; if significant associated p-values are reported in italics; n.s.: not significant.

## Results

### Behavioral Results

#### Task performance during fMRI

Behavioral performances obtained from the experimental fMRI task are summarized in Table 1. The rate of omitted Go trials was alike between the incongruent and congruent stimuli. Analyzing errors during Go trials revealed a significant interaction (F(1,26) = 14.57; p<0.001) of all three main factors entering the analysis of variance (type-by-deadline-by-accuracy), permitting the computation of post-hoc comparisons (right column in Table 1). The rate of erroneous Go responses either within or beyond the response deadline did not differ significantly with respect to stimulus type (congruent, incongruent). However, within the predefined time interval significantly less incongruent than congruent Go trials were correctly executed. Accordingly, the rate of correct Go responses executed beyond the predefined time-window was significantly higher upon incongruent than upon congruent stimuli. Within each stimulus type post-hoc tests (not tabulated in Table 1) also showed that rates of incorrect incongruent Go responses were not significantly different (p = 0.366) with respect to the factor response deadline (6.2 vs. 4.8), as were incorrect congruent Go responses (p = 0.165; 6.3 vs. 3.1). Finally, incorrect responses on NoGo trials were significantly more often committed on incongruent than on congruent stimulus arrays. From Table 1 it also becomes evident (see values in squared brackets) that only for incongruent NoGo trials all 27 participants committed errors.

#### Psychometric results

Putative violations of orthogonality between scales were tested by computing the inter-correlations between individual NEO-PI-R domain scores. Results on inter-correlations of NEO-PI-R scores are summarized in Table 2. Neuroticism was significantly negatively correlated with Extraversion and Conscientiousness. Furthermore, Extraversion was significantly and positively correlated with Openness to Experience. To control for possible associations with age this independent variable was additionally added to the computation of correlations, however revealing no significant correlation coefficients with either scale.

In a next step, we tested on significant correlations between the NEO-PI-R major domain scores and error rates from the fMRI task. Since we considered personality traits to be represented on a superordinate level, observed correlations were only treated as substantial if they showed statistical significance for both types of stimulus arrays (congruent/incongruent), or by demonstrating at least a trend to significance (p-values below 0.1) for one of the stimulus types when the other was significant. We observed that Conscientiousness was significantly negatively correlated with mean percentage of incongruent Go omissions (r = −0.45, p = 0.018), and negatively correlated with mean percentage of congruent Go omissions with a trend to significance (r = −0.35, p = 0.073). Neuroticism was positively correlated with omissions on congruent (r = 0.41, p = 0.035) and incongruent (r = 04.2, p = 0.028) Go trials. The same procedure was applied for correlations between erroneous reaction times (data not shown) and the NEO-PI-R scores. According to our predefined criterion, the only meaningful correlation was observed for Conscientiousness. The higher individuals scored on this scale the slower were correct reaction times for incongruent (r = 0.67; p = 0.006) and congruent (r = 0.64; p = 0.007) Go trials. Correlation coefficients with Neuroticism were again in the opposite direction, although only the correlation with correct reaction times on congruent Go trials (r = −0.55, p = 0.029) was significant, while it was not for incongruent Go trials (r = −0.40; p = 0.121).

### Functional Imaging Results

#### Main effect analyses for error processing during incongruent NoGo trials

**Figure 2:**
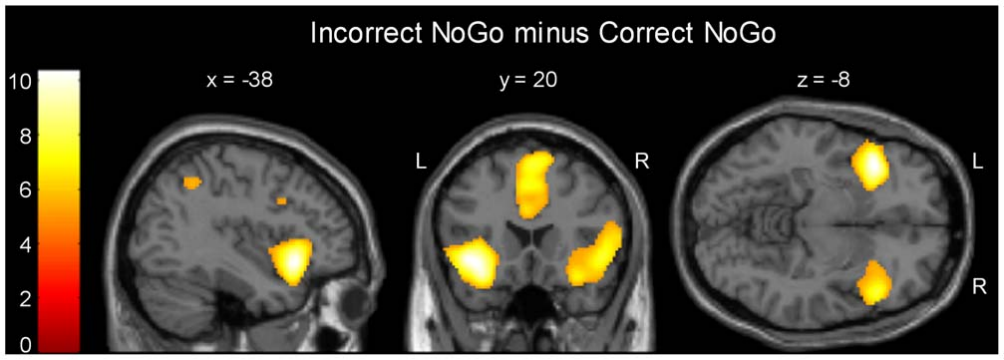
Main effects of error signaling for incongruent NoGo trials. Main effects are reported at a level of *p*<0.025 to account for the one-sidedness of the directed t-contrast, and family-wise (FWE) corrected at the voxel level to control for multiple comparisons (see also Table 3).

Analyzing the main effect of error processing using the contrast of neural activities associated with incorrect and correct NoGo trials, we observed significant (p<0.025, FWE corrected) cortical effects in the left ACC (Brodmann Area [BA] 32), left and right inferior frontal gyrus (BA 47) and adjacent anterior insula, left and right superior parietal lobule (BA 40), and left and right middle frontal gyrus (BA 46/9) (see Figure 2). Subcortically, the contrast revealed significant error signals in right cerebellum and left thalamus. A summary of MNI-coordinates of peak voxels and cluster sizes is given in Table 3. In the reverse contrast (correct minus incorrect NoGo responses), we did not find any significant differences even when lowering the significance to a level of p<0.01 (uncorrected).

**Table 3:** Main effects of error signaling for incongruent NoGo trials.

*Anatomical Region*	BA	*x*	*y*	*z*	*Z*	c
Left inferior frontal gyrus/anterior insula	47	−38	20	−8	7.47	1791
Left anterior cingulate cortex/medial	32/9	6	32	28	6.65	3393
superior frontal gyrus						
Left inferior parietal lobule	40	−62	−46	38	6.73	1407
Right inferior frontal gyrus/anterior insula	47	50	22	−6	6.60	1438
Right inferior parietal lobule	40	54	−38	50	5.72	918
Left middle frontal gyrus	9	−46	10	32	5.19	154
Right middle frontal gyrus	46	48	38	22	5.06	57
Right middle frontal gyrus	9	42	12	38	5.05	69
Right cerebellum	-	22	−54	−28	4.84	23
Left thalamus	-	−10	−14	−4	4.71	16

Main effects are reported at a level of *p*<0.025 to account for the one-sidedness of the directed t-contrast, and family-wise (FWE) corrected at the voxel level to control for multiple comparisons. x, y and z are MNI coordinates of the peak voxel within a cluster. Z: z-value of standard normal distribution; BA = Brodmann area; c: activation cluster sizes, in voxels.

#### Correlations between error activity and personality traits

**Figure 3:**
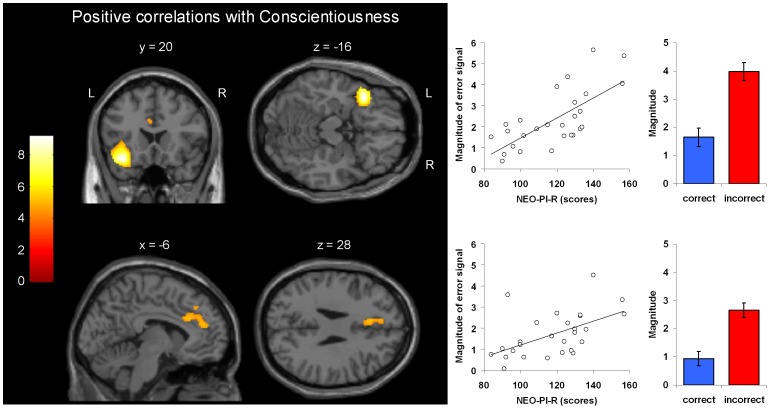
Positive correlations with Conscientiousness in the left inferior frontal gyrus bordering the anterior insula and in ACC reaching into the medial superior frontal gyrus. Correlation coefficients were computed within an inclusive mask consisting of voxels with significant (p<0.025, family-wise corrected) error signaling during incongruent NoGo trials (see also Table 4.).

A first-step multiple regression analysis with all five scales and age included did not show any significant correlation (F-test; p<0.001) of brain activity with individual expressions of subscales Agreeableness, Extraversion, Openness and age. Significant effects were observed for Conscientiousness and Neuroticism. Therefore, in a second-step, the same multiple regression analysis was repeated however now excluding the insignificant sub-scales and age. A summary of results is presented in Table 4. This analysis demonstrated significant positive correlations with Conscientiousness in the left inferior frontal gyrus bordering the anterior insula and in ACC reaching into the medial superior frontal gyrus (see Figure 3). Only positive correlations were observed for this scale. For Neuroticism one cluster of voxels with negative correlations emerged in the left inferior frontal cortex, at the same anatomical location where positive correlations were observed for Conscientiousness.

**Table 4:** Summary of significant positive and negative correlations between NoGo error signaling and NEO-PI-R major domain scores obtained from the reduced model after excluding scales Agreeableness, Extraversion and Openness not showing any significant correlations in the full model.

	Anatomical Region	x/y/z	z-value	Pv(FWE)	extent	Pc(FWE)	Partial R^2^
**Conscientiousness**	Left inferior frontal gyrus (BA47)/	−38/20/−16	6.02	<0.001	996	<0.001	0.49
	Left anterior insula	−54/24/−4	3.78	0.040			
	Anterior cingulate gyrus (BA32)/	−6/26/28	4.18	0.011	260	<0.001	0.29
	Medial superior frontal gyrus (BA 9)	−6/4420	4.15	0.012			
**Neuroticism**	Left inferior frontal gyrus (BA47)	−38/18/−18	3.76	0.028	15	0.021	0.46

Correlation coefficients were computed within an inclusive mask consisting of voxels with significant (p<0.025, family-wise corrected) error signaling during incongruent NoGo trials; x,/y/z: MNI-coordinates of the significant peak voxel in correlation analyses; Pv(FEW) indicates family-wise corrected significance of peak voxel; extent: number of significant (p<0.05, FWE) voxels; Pc(FWE): associated family-wise corrected p-value at the cluster-level; Partial R^2^: cluster averaged partial determination coefficient.

## Discussion

The goal of the present study was to investigate the association of the Five Factor Model's personality traits with neural correlates of error signaling obtained during a combined Eriksen Flanker/GoNoGo task within an event-related fMRI design. Behavioral data analyses showed that subjects indexed sufficient task engagement, as reflected by significantly different error rates depending on the factor stimulus type (congruent/incongruent). Errors of omission were significantly negatively correlated with Conscientiousness. Significant positive correlations with this scale were observed for correct reaction times on Go trials. None of the traits showed any significant correlation with incongruent NoGo errors supporting the note that the functional imaging correlations between personality traits and error signals for this condition are not likely to be confounded by already pre-existing correlations between personality traits and task performance [39].

Functionally, we identified the ACC (BA 32) and the inferior frontal cortex (IFC, BA 47) involved in error monitoring. These areas are in good accordance with previous reports (see Introduction) considered to form the core neural error-related network in humans. Also in line with previous findings is our result of increased insula activation in response to errors [40], [41]. Recently, it was suggested that insula activity may reflect autonomic arousal associated with error signals [11]. Since some of the sub-scales of the NEO-PR-R were significantly inter-correlated to various degrees, correlation analyses between personality traits and error signaling was computed within a two-step multiple regression model testing on significant effects for each scale beyond and above the remaining scales. Individual expressions of Conscientiousness were positively correlated with error signaling in the left IFC adjoining the anterior insula and in the ACC. Neuroticism showed a negative correlation with error signals in the left IFC.

### Conscientiousness

The strongest results of the present study were observed for Conscientiousness yielding exclusively positive correlation coefficients with error signaling in two brain regions, the ACC and left inferior frontal gyrus (IFG) with adjacent anterior insula (IFG/aI).

Recently, Tops and Boksem [42] have suggested the IFG/aI to be part of a ventrolateral corticolimbic pathway that implements control over actions through higher responsiveness to momentary environmental stimuli. Similarly, previous work on cognitive control of memory suggests that the left IFG is involved in the control of goal or task-related memory representations [43], [44], [45] enabling strategic handling of task-related memory representations in order to meet goals at higher levels. This interpretation is in line with a recent meta-analysis of 10 different fMRI tasks [20] that were analyzed regarding task-set specific processes defined as task-set initiation activity, task-set maintenance activity, and error-related task-set activity. Besides the dorsal ACC bordering the medial superior frontal cortex, the IFG and anterior insula were summarized to form the core of a human task-set system. This task-set system is considered to monitor bottom-up signals of ongoing performance, indicating discrepancies about the intended and actual outcome. The system either reflects or generates error-related signals that in turn help to adjust ensuing top-down signals in service of ensuing error processing. Given this empirically based theoretical account of the inferior frontal involvement during error signaling, it supports the idea that enhancement of error signals may likely reflect a neural representation of an error-related violation of the task-set system. The strong correlation between error signaling and Conscientiousness in the left IFG/aI aligns with this interpretation since this trait is characterized by a strong goal-directed behavior. Conscientious individuals focus on task accomplishment and fulfillment of obligations and also load high on achievement striving, which reflects individual's intention to accomplish given tasks expressed as a high goal orientation. Hence, modulating effects of Conscientiousness on neural error processing in the IFC may reflect the degree to which individuals' goal direction is violated by the occurrence of errors.

Involvement of the dorsal ACC has commonly been reported in studies on the neural correlates of error processing and functional interpretation depends on theoretical background. These theoretical accounts contrast pure error detection [46], [47] with the identification of response conflict [17], [48], [49], [50] that arises when two competing responses are simultaneously activated. More recent proposals suggest errors to represent a special case of higher order conflict processing [13], [51]. In the context of reinforcement learning [10], errors are interpreted as negative events, and differentially increased activation of the ACC upon errors may reflect more than just a cognitive process, suggesting an evaluative function for enhanced ACC signaling in a sense that this structure is more generally responsive to negatively valenced signals that may arise from error or conflict monitoring [52]. Accordingly, the strong correlation with Conscientiousness may therefore reflect a trait dependent modulation of this negatively valenced signal. The more conscientious (focusing on task accomplishment) subjects are, the more they experience violation of goal directions as a negative signal, either by the occurrence of errors per se or by the conflict that arises thereof.

### Neuroticism

The negative correlation between error signalling and Neuroticism was opposite to the direction observed for Conscientiousness, although in a corresponding anatomical location. Statistically, the opposite direction of correlation coefficients appears to be a direct expression of the negative correlation between both scales observed from correlation analysis of questionnaire scores in our sample. Thus, statistically the inter-correlation between Neuroticism and Conscientiousness has contributed to the negative correlation with Neuroticism in the IFC. This is further supported by the opposite correlations of both scales with behavioral measures obtained from the fMRI task (omission errors on Go trials; reaction time for correct Go trials). Already Costa and McCrae [1] have reported that Neuroticism and Conscientiousness were negatively related (r = −.53) in their validation study of the NEO-PI-R, and also other studies have observed negative correlations between these two traits [30], [53].

The analyses of functional MRI data show that correlations of both scales with error signalling appear within the same anatomical location. Previous investigations have already linked Conscientiousness and Neuroticism to intentional involvement in opposite directions. In these studies Conscientiousness has been shown to be less sensitive to motivational manipulations, while Neuroticism appeared to modulate neural processing in the presence of rewards [30], [54]. Therefore, assuming that these two traits relate differently to task engagement, and interpreting errors to signal the absence of expected rewards, the present correlation with Neuroticism suggests that a decreasing error signal with increasing Neuroticism emerges since more neurotic individuals may have greater propensity to fade out the absence of reward. This is opposite to less reward-modulated conscientious individuals with higher intrinsic task engagement per se. Still, this interpretation awaits replication in samples with less negative inter-correlation between Conscientiousness and Neuroticism.

### Absence of significant correlations with Extraversion

Contrary to our expectation, a correlation between Extraversion and neural error signaling was not observed under present statistical thresholds. This correlation was particularly expected to appear in the ACC due to the strong relations of this trait with reward sensitivity and due to the theoretical accounts suggesting error signals to reflect a negative reinforcement learning signal which is conveyed by the ACC (e.g. [55]; see also below). Instead, strong positive correlations in this anatomical structure emerged for Conscientiousness. Having used a multiple regression analysis, this observation suggests that Conscientiousness may have absorbed a crucial part of individual variances of error signaling in the ACC, so that only a smaller part of unexplained variance remained to be explained by Extraversion. To test this prediction an exploratory analysis of the full model with decreased statistical thresholds (data not shown) indeed yielded voxels in the ACC bearing positive correlation coefficients with Extraversion at corresponding locations where Conscientiousness produced higher and more reliable coefficients. Accordingly, in a second exploratory analysis using a simple regression model by leaving out all the other scales, correlation coefficients with Extraversion in the ACC markedly increased, however did still not survive the a priori set statistical threshold of p<0.05, family-wise corrected.

This results pattern supports that a focus on a single, pre-selected personality trait might be misleading when exploring the influence of individual differences. Although Extraversion and Conscientiousness in our present sample were only slightly positively correlated (r = 0.14), which was by far not significant, a restriction to one or the other scale would have led to different interpretations. Neither of these interpretations would have been entirely wrong, since both scales associated positively with error signaling in a process relevant brain structure, however at markedly different levels of significance. Given the still exploratory aspect of this and other studies that try to elucidate the neurobiological implications of personality, adherence to a conservative and invariant statistical threshold appears mandatory, though. Beyond mere statistical reasoning, the greater reliability observed for Conscientiousness may also indicate greater construct validity for this scale than for Extraversion given the process of interest.

### Limitations

Several aspects of this study appear noteworthy as limiting factors. As with other correlating studies, we want to remind that present modulating effects of Neuroticism and especially Conscientiousness on neural error signaling are based on a pure correlation approach which can merely identify those relationships between variables as they were measured. Therefore, we cannot rule out that present associations may have been conditioned by one or more yet unknown other factors. Although a larger sample of 27 subjects was included in the present study, sample size remains an issue that must be considered in correlation studies, especially when discussing the absence of significant effects. This absence might change with inclusion of even more subjects. Finally, the present fMRI task incorporated feedback about the correctness of subjects' responses in very close temporal succession to the stimulus arrays, which cannot be resolved by the comparably slow hemodynamic response function. Although inclusion of feedback appears reasonable in a task on error monitoring to counteract any ambiguities of subjects' individual responses, its influence on present results could not be controlled. However, an exploratory analysis modelling onsets of feedback instead of onsets of stimulus arrays did not change results when contrasting incorrect minus correct incongruent NoGo trials to delineate brain regions reliably associated with error signalling.

### Conclusion

We explored the modulating influences of the Five Factor Model's personality dispositions on neural error signalling, and Conscientiousness as a trait explained the greatest part of individual differences in neural activities associated with error monitoring. Given the psychological mechanisms reflected by this trait, the putative functional roles of those differentially activated brain regions, and the highly significant positive correlations, our results lend further external validity to this specific personality trait suggesting that it does reflect more than being a mere descriptive taxonomy. Although interpretation of the absence of significant effects is generally to be treated with caution, Openness to experience and Agreeableness did not explain relevant parts of individual variance in error signalling. However, at least for the latter trait this result appears plausible in terms of discriminant validity, since Agreeableness has been related to psychological mechanisms involved in social information processing which was not tested by the present task. Neuroticism was negatively correlated with error signalling, possibly reflecting the negative inter-correlation between this scale and Conscientiousness already being observed on the behavioral level. Finally, correlations between Extraversion and error signalling did not survive present statistical thresholds although some additional exploratory analyses could not entirely rule out its modulating effects on individual neural activities of error monitoring brain areas.

Taken together present results illustrate that for predicting individual responses to errors underlying personality traits should be taken into account. Although a reliable observation of error signalling at the group level was not affected by individual expressions of traits under investigation, future research should consider the modulating effects of personality to advance the neurobiological implications of individual differences in this and other cognitive domains.

## Supporting Information

Supporting Information S1 **reports results on Go errors (stemming from the contrast of false minus correct Go responses), conjoint effects of NoGo and Go errors, and a summary of significant positive and negative correlations between the Go error signaling and NEO-PI-R major domain scores Conscientiousness and Neuroticism obtained from the reduced model after excluding the scales Agreeableness, Extraversion and Openness not showing any significant correlations in the full model.**
(DOC)

## References

[1] Costa PT Jr, McCrae RR (1997) Stability and change in personality assessment: the revised NEO Personality Inventory in the year 2000. J Pers Assess 68: 86–94. 9018844 10.1207/s15327752jpa6801_7

[2] Canli T (2004) Functional brain mapping of extraversion and neuroticism: learning from individual differences in emotion processing. J Pers 72: 1105–1132. 15509278 10.1111/j.1467-6494.2004.00292.x

[3] Omura K, Todd Constable R, Canli T (2005) Amygdala gray matter concentration is associated with extraversion and neuroticism. Neuroreport 16: 1905–1908. 16272876 10.1097/01.wnr.0000186596.64458.76

[4] Cremers H, van Tol MJ, Roelofs K, Aleman A, Zitman FG, et al (2011) Extraversion is linked to volume of the orbitofrontal cortex and amygdala. PLoS One 6: e28421. 22174802 10.1371/journal.pone.0028421 PMC3235124

[5] DeYoung CG, Hirsh JB, Shane MS, Papademetris X, Rajeevan N, et al (2010) Testing predictions from personality neuroscience. Brain structure and the big five. Psychol Sci 21: 820–828. 20435951 10.1177/0956797610370159 PMC3049165

[6] Adelstein JS, Shehzad Z, Mennes M, Deyoung CG, Zuo XN, et al (2011) Personality is reflected in the brain's intrinsic functional architecture. PLoS One 6: e27633. 22140453 10.1371/journal.pone.0027633 PMC3227579

[7] Canli T, Sivers H, Whitfield SL, Gotlib IH, Gabrieli JD (2002) Amygdala response to happy faces as a function of extraversion. Science 296: 2191. 12077407 10.1126/science.1068749

[8] Haas BW, Constable RT, Canli T (2008) Stop the sadness: Neuroticism is associated with sustained medial prefrontal cortex response to emotional facial expressions. Neuroimage 42: 385–392. 18511299 10.1016/j.neuroimage.2008.04.027 PMC2789588

[9] Cremers HR, Demenescu LR, Aleman A, Renken R, van Tol MJ, et al (2010) Neuroticism modulates amygdala-prefrontal connectivity in response to negative emotional facial expressions. Neuroimage 49: 963–970. 19683585 10.1016/j.neuroimage.2009.08.023

[10] Holroyd CB, Coles MG (2002) The neural basis of human error processing: reinforcement learning, dopamine, and the error-related negativity. Psychol Rev 109: 679–709. 12374324 10.1037/0033-295X.109.4.679

[11] Ullsperger M, Harsay HA, Wessel JR, Ridderinkhof KR (2010) Conscious perception of errors and its relation to the anterior insula. Brain Struct Funct 214: 629–643. 20512371 10.1007/s00429-010-0261-1 PMC2886909

[12] Rodriguez-Fornells A, Kurzbuch AR, Munte TF (2002) Time course of error detection and correction in humans: neurophysiological evidence. J Neurosci 22: 9990–9996. 12427856 10.1523/JNEUROSCI.22-22-09990.2002 PMC6757828

[13] Taylor SF, Stern ER, Gehring WJ (2007) Neural systems for error monitoring: recent findings and theoretical perspectives. Neuroscientist 13: 160–172. 17404376 10.1177/1073858406298184

[14] Herrmann MJ, Rommler J, Ehlis AC, Heidrich A, Fallgatter AJ (2004) Source localization (LORETA) of the error-related-negativity (ERN/Ne) and positivity (Pe). Brain Res Cogn Brain Res 20: 294–299. 15183400 10.1016/j.cogbrainres.2004.02.013

[15] Ridderinkhof KR, Ullsperger M, Crone EA, Nieuwenhuis S (2004) The role of the medial frontal cortex in cognitive control. Science 306: 443–447. 15486290 10.1126/science.1100301

[16] Ridderinkhof KR, van den Wildenberg WP, Segalowitz SJ, Carter CS (2004) Neurocognitive mechanisms of cognitive control: the role of prefrontal cortex in action selection, response inhibition, performance monitoring, and reward-based learning. Brain Cogn 56: 129–140. 15518930 10.1016/j.bandc.2004.09.016

[17] Ullsperger M, von Cramon DY (2001) Subprocesses of performance monitoring: a dissociation of error processing and response competition revealed by event-related fMRI and ERPs. Neuroimage 14: 1387–1401. 11707094 10.1006/nimg.2001.0935

[18] Braver TS, Barch DM, Gray JR, Molfese DL, Snyder A (2001) Anterior cingulate cortex and response conflict: effects of frequency, inhibition and errors. Cereb Cortex 11: 825–836. 11532888 10.1093/cercor/11.9.825

[19] Debener S, Ullsperger M, Siegel M, Fiehler K, von Cramon DY, et al (2005) Trial-by-trial coupling of concurrent electroencephalogram and functional magnetic resonance imaging identifies the dynamics of performance monitoring. J Neurosci 25: 11730–11737. 16354931 10.1523/JNEUROSCI.3286-05.2005 PMC6726024

[20] Dosenbach NU, Visscher KM, Palmer ED, Miezin FM, Wenger KK, et al (2006) A core system for the implementation of task sets. Neuron 50: 799–812. 16731517 10.1016/j.neuron.2006.04.031 PMC3621133

[21] Garavan H, Ross TJ, Murphy K, Roche RA, Stein EA (2002) Dissociable executive functions in the dynamic control of behavior: inhibition, error detection, and correction. Neuroimage 17: 1820–1829. 12498755 10.1006/nimg.2002.1326

[22] Mathalon DH, Whitfield SL, Ford JM (2003) Anatomy of an error: ERP and fMRI. Biol Psychol 64: 119–141. 14602358 10.1016/s0301-0511(03)00105-4

[23] Menon V, Adleman NE, White CD, Glover GH, Reiss AL (2001) Error-related brain activation during a Go/NoGo response inhibition task. Hum Brain Mapp 12: 131–143. 11170305 10.1002/1097-0193(200103)12:3<131::AID-HBM1010>3.0.CO;2-C PMC6872006

[24] Dikman ZV, Allen JJ (2000) Error monitoring during reward and avoidance learning in high- and low-socialized individuals. Psychophysiology 37: 43–54. 10705766

[25] Ruchsow M, Spitzer M, Gron G, Grothe J, Kiefer M (2005) Error processing and impulsiveness in normals: evidence from event-related potentials. Brain Res Cogn Brain Res 24: 317–325. 15993769 10.1016/j.cogbrainres.2005.02.003

[26] Hajcak G, McDonald N, Simons RF (2004) Error-related psychophysiology and negative affect. Brain Cogn 56: 189–197. 15518935 10.1016/j.bandc.2003.11.001

[27] Compton RJ, Carp J, Chaddock L, Fineman SL, Quandt LC, et al (2007) Anxiety and error monitoring: increased error sensitivity or altered expectations? Brain Cogn 64: 247–256. 17482740 10.1016/j.bandc.2007.03.006 PMC1995669

[28] Olvet DM, Hajcak G (2011) The error-related negativity relates to sadness following mood induction among individuals with high neuroticism. Soc Cogn Affect Neurosci 10.1093/scan/nsr007 PMC3304478 21382967

[29] Tops M, Boksem MA (2010) Absorbed in the task: Personality measures predict engagement during task performance as tracked by error negativity and asymmetrical frontal activity. Cogn Affect Behav Neurosci 10: 441–453. 21098805 10.3758/CABN.10.4.441

[30] Pailing PE, Segalowitz SJ (2004) The error-related negativity as a state and trait measure: motivation, personality, and ERPs in response to errors. Psychophysiology 41: 84–95. 14693003 10.1111/1469-8986.00124

[31] Cloninger CR (1986) A unified biosocial theory of personality and its role in the development of anxiety states. Psychiatr Dev 4: 167–226. 3809156

[32] Eysenck HJ (1981) General features of the model. In: Eysenck HJ, editor. A model for personality. 1st ed. Berlin: Springer. pp. 1–37.

[33] Horn NR, Dolan M, Elliott R, Deakin JF, Woodruff PW (2003) Response inhibition and impulsivity: an fMRI study. Neuropsychologia 41: 1959–1966. 14572528 10.1016/s0028-3932(03)00077-0

[34] Graf H, Abler B, Freudenmann R, Beschoner P, Schaeffeler E, et al (2011) Neural correlates of error monitoring modulated by atomoxetine in healthy volunteers. Biol Psychiatry 69: 890–897. 21168122 10.1016/j.biopsych.2010.10.018

[35] Vasic N, Plichta MM, Wolf RC, Fallgatter AJ, Sosic-Vasic Z, et al.. (2012) Reduced neural error signaling in left inferior prefrontal cortex in young adults with ADHD. Journal of Attention Disorders: in press. 10.1177/1087054712446172 22660917

[36] Oldfield RC (1971) The assessment and analysis of handedness: the Edinburgh inventory. Neuropsychologia 9: 97–113. 5146491 10.1016/0028-3932(71)90067-4

[37] Borkenau P, Ostendorf F (2008) [NEO-Five-Factors Inventory, Costa and McCrae (NEO-FFI), a manual. Göttingen: Hogrefe.

[38] Eriksen BA, Eriksen CW (1974) Effects of noise letters upon the identification of a target letter in a nonsearch task. Percept Psychophys 16: 143–149.

[39] Price CJ, Friston KJ (1999) Scanning patients with tasks they can perform. Hum Brain Mapp 8: 102–108. 10524600 10.1002/(SICI)1097-0193(1999)8:2/3<102::AID-HBM6>3.0.CO;2-J PMC6873312

[40] Garavan H, Ross TJ, Kaufman J, Stein EA (2003) A midline dissociation between error-processing and response-conflict monitoring. Neuroimage 20: 1132–1139. 14568482 10.1016/S1053-8119(03)00334-3

[41] Hester R, Garavan H (2004) Executive dysfunction in cocaine addiction: evidence for discordant frontal, cingulate, and cerebellar activity. J Neurosci 24: 11017–11022. 15590917 10.1523/JNEUROSCI.3321-04.2004 PMC6730277

[42] Tops M, Boksem MA (2011) A potential role of the inferior frontal gyrus and anterior insula in cognitive control, brain rhythms, and event-related potentials. Front Psychol 2: 330. 22084637 10.3389/fpsyg.2011.00330 PMC3212750

[43] Badre D, Wagner AD (2005) Frontal lobe mechanisms that resolve proactive interference. Cereb Cortex 15: 2003–2012. 15788702 10.1093/cercor/bhi075

[44] Badre D, Wagner AD (2007) Left ventrolateral prefrontal cortex and the cognitive control of memory. Neuropsychologia 45: 2883–2901. 17675110 10.1016/j.neuropsychologia.2007.06.015

[45] Wagner AD, Maril A, Bjork RA, Schacter DL (2001) Prefrontal contributions to executive control: fMRI evidence for functional distinctions within lateral Prefrontal cortex. Neuroimage 14: 1337–1347. 11707089 10.1006/nimg.2001.0936

[46] Coles MG, Scheffers MK, Holroyd CB (2001) Why is there an ERN/Ne on correct trials? Response representations, stimulus-related components, and the theory of error-processing. Biol Psychol 56: 173–189. 11399349 10.1016/s0301-0511(01)00076-x

[47] Falkenstein M, Hohnsbein J, Hoormann J, Blanke L (1991) Effects of crossmodal divided attention on late ERP components. II. Error processing in choice reaction tasks. Electroencephalogr Clin Neurophysiol 78: 447–455. 1712280 10.1016/0013-4694(91)90062-9

[48] Carter CS, Braver TS, Barch DM, Botvinick MM, Noll D, et al (1998) Anterior cingulate cortex, error detection, and the online monitoring of performance. Science 280: 747–749. 9563953 10.1126/science.280.5364.747

[49] Kerns JG, Cohen JD, Stenger VA, Carter CS (2004) Prefrontal cortex guides context-appropriate responding during language production. Neuron 43: 283–291. 15260963 10.1016/j.neuron.2004.06.032

[50] Yeung N, Botvinick MM, Cohen JD (2004) The neural basis of error detection: conflict monitoring and the error-related negativity. Psychol Rev 111: 931–959. 15482068 10.1037/0033-295x.111.4.939

[51] Botvinick MM, Braver TS, Barch DM, Carter CS, Cohen JD (2001) Conflict monitoring and cognitive control. Psychol Rev 108: 624–652. 11488380 10.1037/0033-295x.108.3.624

[52] Aston-Jones G, Cohen JD (2005) An integrative theory of locus coeruleus-norepinephrine function: adaptive gain and optimal performance. Annu Rev Neurosci 28: 403–450. 16022602 10.1146/annurev.neuro.28.061604.135709

[53] Saucier G, Goldberg LR, Institute OR (2001) Lexical studies of indigenous personality factors: premises, products, and prospects. J Pers 69: 847–879. 11767821 10.1111/1467-6494.696167

[54] Tops M, Boksem MA, Wester AE, Lorist MM, Meijman TF (2006) Task engagement and the relationships between the error-related negativity, agreeableness, behavioral shame proneness and cortisol. Psychoneuroendocrinology 31: 847–858. 16774808 10.1016/j.psyneuen.2006.04.001

[55] Holroyd CB, Yeung N (2012) Motivation of extended behaviors by anterior cingulate cortex. Trends Cogn Sci 16: 122–128. 22226543 10.1016/j.tics.2011.12.008

