# Supplementary material for: The Modulating Effect of Personality Traits on Neural Error Monitoring: Evidence from Event-Related fMRI
Source: PLoS One. 2012 Aug 22;7(8):e42930. doi: 10.1371/journal.pone.0042930 (PMC3425580; doi:10.1371/journal.pone.0042930)
Supplement: Supporting Information S1 — reports results on Go errors (stemming from the contrast of false minus correct Go responses), conjoint effects of NoGo and Go errors, and a summary of significant positive and negative correlations between the Go error signaling and NEO-PI-R major domain scores Conscientiousness and Neuroticism obtained from the reduced model after excluding the scales Agreeableness, Extraversion and Openness not showing any significant correlations in the full model. (DOC) [file pone.0042930.s001.doc]

**The Modulating Effect of Personality Traits on Neural Error Processing: Evidence from Event-Related fMRI**

Zrinka Sosic-Vasic, Martin Ulrich, Martin Ruchsow, Nenad Vasic, Georg Grön

- Supporting Information-

In the present study we were using a combined Eriksen-Flanker/GoNogo-task with two modifications added to the previous version of this task. First, in order to emphasize the speed component during task performance a response deadline was additionally inserted in case of Go responses. With the intention that only correct and false Go responses within individually predefined time windows should be used to estimate the error signal, the risk of potential confounds due to an unbalanced speed-accuracy should be minimized by this modification. Secondly, for the Go condition a two-alternative choice reaction was inserted in order to realize that second source of errors beyond erroneous NoGo responses (errors of commission) which should permit control of motor components in case of NoGo responses. By definition the neural error signal during NoGo responses is estimated by the contrast of false minus correct NoGo responses. Since the former responses posses a motor reaction component, the error signal from the contrast above could be confounded by this component. In case of Go errors stemming from the contrast of false minus correct Go responses the motor component in both responses is equivalent. This additional error signal was intended to be used to control for the motor component in case of NoGo errors.

Despite these modifications above error rates on Go responses were rather low with on average five trials per subject for the incongruent stimulus arrays. For congruent arrays some of our subjects did not make an error at all. Consequently, the individual error signals on Go errors were inappropriate for correlation analyses with individual expressions of personality traits. Nevertheless, at the group level we made use of Go errors on incongruent stimulus arrays in order to estimate at least the neural structures associated with the processing of this specific error and how this error matrix compares to that associated with NoGo errors, finally in order to control for the motor component inherent in NoGo errors which is subtracted out using Go errors (see above).

Therefore, for both conditions (incongruent NoGo and incongruent Go) individual contrast images were computed by subtracting estimated neural activity associated with incorrect minus correct responses. These individual contrast images were then propagated to a random effects model to average each main effect (NoGo errors and Go errors) separately (see Table S1) as well as a conjunction thereof, however at a slightly more lenient level of significance (p < 0.0005 uncorrected at the voxel level, and p < 0.05, corrected at a cluster level (minimum of 321 conjointly significant voxels per cluster)) which then served as an explicit inclusive mask for correlation analyses of NoGo errors with personality traits in the same way as reported in the main paper.

Anatomical regions bearing significant main effects for each error type are summarized in Table S1 (no significant results for the inverted contrast). Overall, contrasting incorrect minus correct Go responses led to a less extended error matrix than that observed for NoGo errors although a subset of anatomical regions showed considerable overlap. This was explicitly tested by a conjunction analysis of both contrasts for NoGo and Go trials and anatomical regions bearing a significant conjoined error signal are also summarized in Table S1 (lower part) and are depicted in Figure S1.

**Table S1. Main effects of NoGo and Go error processing and their conjunction.**

| ***Anatomical Region*** | ***BA*** | ***x*** | ***y*** | ***z*** | ***Z*** | **c** |
| --- | --- | --- | --- | --- | --- | --- |
| **NoGo incorrect minus NoGo correct** | | | | | |  |
| Left inferior frontal gyrus/anterior insula | 47 | -38 | 20 | -8 | 7.47 | 1791 |
| Left anterior cingulate cortex/medial frontal gyrus | 32/9 | 6 | 32 | 28 | 6.65 | 3393 |
| Left inferior parietal lobule | 40 | -62 | -46 | 38 | 6.73 | 1407 |
| Right inferior frontal gyrus/anterior insula | 47 | 50 | 22 | -6 | 6.60 | 1438 |
| Right inferior parietal lobule | 40 | 54 | -38 | 50 | 5.72 | 918 |
| Left middle frontal gyrus | 9 | -46 | 10 | 32 | 5.19 | 154 |
| Right middle frontal gyrus | 46 | 48 | 38 | 22 | 5.06 | 57 |
| Right middle frontal gyrus | 9 | 42 | 12 | 38 | 5.05 | 69 |
| Right cerebellum | - | 22 | -54 | -28 | 4.84 | 23 |
| Left thalamus | - | -10 | -14 | -4 | 4.71 | 16 |
| **Go incorrect minus Go correct** | | | | | |  |
| Left inferior frontal gyrus | 47 | -44 | 22 | 0 | 5.37 | 112 |
| Left inferior parietal gyrus | 40 | -62 | -46 | 38 | 5.15 | 73 |
| Left superior frontal gyrus | 46 | -28 | 56 | 22 | 5.14 | 42 |
| Left supplementary motor area | 6 | -6 | 16 | 62 | 5.03 | 60 |
| Left anterior cingulate cortex | 32 | -4 | 24 | 40 | 5.01 | 62 |
| **Conjunction analysis** | | | | | |  |
| Left inferior frontal gyrus | 47 | -44 | 22 | 0 | 5.37 | 916 |
| Left inferior parietal gyrus | 40 | -62 | -46 | 38 | 5.15 | 709 |
| Left anterior cingulate cortex | 32 | -4 | 24 | 40 | 5.01 | 1500 |
| Left supplementary motor area | 6 | -4 | 18 | 62 | 4.92 |  |

Main effects of NoGo and Go errors are reported at a level of *p* < 0.025 family-wise (FWE) corrected at the voxel level to control for multiple comparisons. Effects of the conjunction analysis are reported at a level of p < 0.0005 uncorrected at the voxel level and p < 0.05 corrected at the cluster level (minimum of 321 contiguously significant voxels per cluster). x, y and z are MNI coordinates of the peak voxel within a cluster. BA = Brodmann area; c: cluster sizes


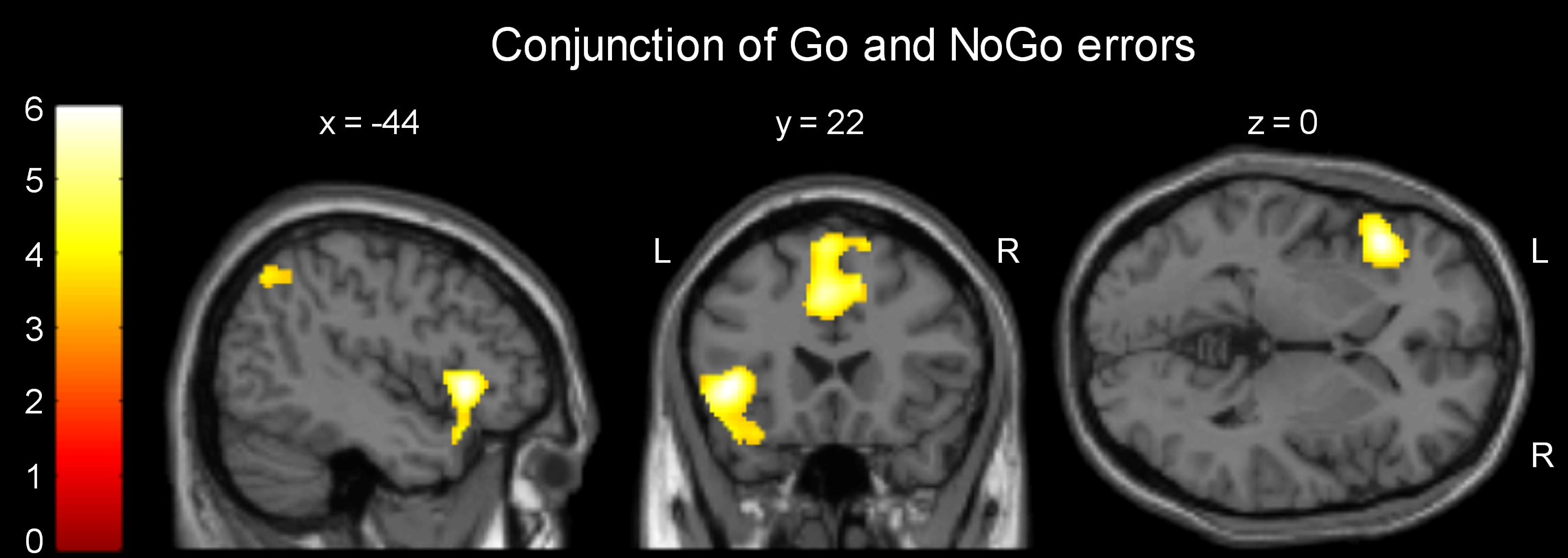


**Figure S1. Conjoint effects of NoGo and Go errors.** Effects of the conjunction analysis of both main effects (p < 0.005 uncorrected at the voxel level; p < 0.05 corrected at the cluster level with a minimum extent of 321 contiguously significant voxels per cluster). For detailed MNI-coordinates and Z-values see Table S1.

Two factors may have contributed to less extend main effects associated with monitoring Go errors. First might be the reduced number of error rates in case of Go trials. Secondly, avoidance of Go errors had not been made explicit to the participants in the instruction to the task while avoidance of NoGo errors had been made explicit. By this instruction-related modification two differentially valenced types of errors may have been induced, namely an explicit and presumably higher valenced error in case of NoGo errors representing the major response goal in our task set, and an implicit and presumably lower valenced error in case of Go errors representing the subordinate goal in the given task set.

Using the statistical map of conjoined significant main effects as an explicit inclusive mask, a multiple regression analysis incorporating the personality traits of Conscientiousness and Neuroticism showed rather the same result pattern as reported in the main paper supporting that the results reported there are most likely not biased by the motor component inherent in erroneous NoGo trials.

|  | **Anatomical Region** | **x/y/z** | **z-value** | **Pv(FWE)** | **c** | **Pc(FWE)** | **Partial R^2^** |
| --- | --- | --- | --- | --- | --- | --- | --- |
| **Conscientiousness** | Left inferior frontal gyrus (BA47)  / left anterior insula | -38/20/-16  -54/26/-4 | 6.02  3.82 | <0.001  0.017 | 414 | <0.001 | 0.55 |
|  | Anterior cingulate gyrus (BA32)/  Medial superior frontal gyrus (BA 9) | -6/26/28  -6/40/26 | 4.18  3.99 | 0.011  0.010 | 402 | <0.001 | 0.27 |
| **Neuroticism** | Left inferior frontal gyrus (BA47) | -38/18/-18 | 3.76 | 0.013 | 28 | 0.015 | 0.42 |

**Table S2**. Summary of significant positive and negative correlations between the Go error signaling and NEO-PI-R major domain scores obtained from the reduced model after excluding scales Agreeableness, Extraversion and Openness not showing any significant correlations in the full model.

Correlation coefficients were computed within an inclusive mask consisting of voxels with a conjointly significant (p<0.0005, uncorrected; cluster extent: 312; p < 0.05, FWE corrected) error signaling during incongruent NoGo and Go trials; x,/y/z: MNI-coordinates of the significant peak voxel in correlation analyses; Pv(FWE) indicates family-wise corrected significance of peak voxel; c: number of significant (p < 0.05, FWE) voxels; Pc(FWE): associated family-wise corrected p-value at the cluster-level; Partial R^2^: partial determination coefficient.
